# A Systematic Review of Innovative Advances in Multi-Material Additive Manufacturing: Implications for Architecture and Construction

**DOI:** 10.3390/ma18081820

**Published:** 2025-04-16

**Authors:** Amirhossein Fakhr Ghasemi, Jose Pinto Duarte

**Affiliations:** Stuckeman Center for Design Computing (SCDC), Stuckeman School of Architecture and Landscape Architecture, Penn State, Stuckeman Family Building, University Park, PA 16802, USA

**Keywords:** additive manufacturing, 3D printing, functionally graded material, function optimization, fused filament fabrication, multi-material additive manufacturing

## Abstract

Additive manufacturing (AM) has made rapid progress in most industries; however, the construction sector lags behind, despite substantial potential for growth. This study aims to evaluate recent innovations in AM, with a focus on multi-material additive manufacturing (MMAM), to identify transferable knowledge and technologies for the construction industry. A systematic Boolean search reviewing the Scopus and Web of Science databases identified 33 relevant articles out of 368 papers published in English over the last five years. Material properties, manufacturing processes, and design approaches were collectively identified as key interdisciplinary factors; these included thermal and mechanical property gradation techniques from materials science, multi-scale optimization approaches from engineering, and real-time monitoring systems from manufacturing, which are each transferable to architectural applications. Bibliometric analysis demonstrated growing research trajectories in AI-driven optimization methods and functionally graded materials that could bridge the implementation gap in construction. This article identifies significant knowledge gaps in scaling laboratory-proven MMAM techniques to architectural applications, including material interface challenges, environmental durability concerns, and the absence of design tools specific to building-scale components. We provide a critical roadmap for researchers that prioritizes the development of integrated optimization frameworks; multiscale modeling techniques; novel material combinations suitable for construction environments; and standardized protocol bases for Equipment Design, Process Control, Design Integration, Digital Tools, and Materials Research for evaluating the long-term performance and safety of MMAM building components.

## 1. Introduction

From creating low-cost housing in impoverished regions to building habitats on other planets, architectural-scale additive manufacturing offers a range of solutions for various applications [1,2]. These applications include deployable military shelters built in as little as 36 h; pedestrian infrastructure such as the world’s longest 3D-printed concrete bridge, in Shanghai; and residential solutions like the Apis Cor House in Russia, constructed in just 24 h using portable 3D printing technology [1].

Additive manufacturing (AM) encompasses a variety of production methods that create physical objects by successively adding material based on a geometrical representation [3,4]. In the architecture and construction industry, AM addresses significant challenges highlighted in the UN’s “Global Sustainable Development Report 2019”, including the need for sustainable building, adaptive urban environments, and improved productivity [5]. In response to these challenges, the industry has increasingly adopted advanced technologies, such as computational design and digital fabrication [6]. Computational design, through parametric modeling and algorithms, enables architects to explore complex geometries while optimizing for multiple performance criteria simultaneously. This method facilitates rapid iteration of design solutions, considering factors like efficiency, environmental impact, and material use [7]. Digital fabrication bridges the gap between complex digital models and physical construction. Technologies like robotic fabrication and 3D printing have expanded the possibilities of architecture, enabling seamless workflows from digital design to building. These advancements have enhanced precision, reduced waste, and offered greater customization and design freedom [8].

Multi-material additive manufacturing (MMAM) represents a major leap forward in 3D printing, allowing the fabrication of complex parts by integrating multiple materials in a single process [7,9]. In the specific context of architecture and construction, MMAM encompasses technologies that enable the strategic spatial distribution of different materials within building components to achieve varied functional properties. This scope includes: (1) the additive fabrication of building elements with discrete material boundaries for multi-functionality, such as the integration of structural and insulating layers within wall sections; (2) the creation of functionally graded materials with continuous property transitions, like gradually changing the density or thermal conductivity across building envelopes; and (3) the incorporation of smart or responsive materials within conventional building components for adaptive performance [2]. This technology holds significant promise for architecture, since buildings must address multiple functional demands while the construction process is tasked with reducing material waste and construction time [2]. While AM is no longer viewed as an academic concept but a pragmatic solution, the architecture, engineering, and construction industry has yet to fully leverage the optimization strategies and advancements seen in other sectors. This paper aims to bridge this gap by reviewing the applications and optimization of MMAM across various disciplines. By drawing on insights from materials science, engineering, and manufacturing, we aim to identify methods that can be adapted to meet the unique needs of architecture. Our goal is to propose ways to apply MMAM in architecture and construction, addressing industry-specific challenges and opportunities with this technology. Through this interdisciplinary approach, we aim to contribute to the development of sustainable, efficient, and innovative building solutions. By studying how MMAM can be harmoniously integrated into architectural frameworks, we aim to facilitate the emergence of buildings that are more responsive and efficient, addressing the diverse demands of our environment.

## 2. Materials and Methods

This review employed a systematic approach to examine optimization techniques in multi-material additive manufacturing and assess their potential application in the architecture, engineering and construction industry. The methodology followed a two-stage process: first, a targeted search focused on material extrusion techniques, followed by a broader inclusion of relevant papers.

### 2.1. Database Selection and Search Strategy

We utilized two scientific databases: Scopus and Web of Science, chosen for their comprehensive coverage of the peer-reviewed literature in engineering, materials science, manufacturing, and construction. The initial search query was as follows: “Additive manufacturing”, “3D printing”, “rapid prototyping”, and “direct digital manufacturing” (These terms are often used in literature to refer to the overall process of layer-by-layer fabrication).

“Functionally graded materials (FGM)”, “gradient materials”, and “multi material” were used to specifically target studies dealing with the combination and spatial variation of multiple materials within a single AM process. “Process optimization”, “function optimization”, “functional optimization”, “performance optimization”, “mechanical optimization”, “thermal optimization”, and “optimization” covered the wide range of optimization objectives and techniques employed in MMAM research. “Fused deposition modeling (FDM)”, “fused filament fabrication (FFF)”, “material extrusion”, and “layer extrusion printing” are all terms referring to the MMAM process, which was the primary focus of this review. Including these synonyms ensured comprehensive coverage of FDM/FFF-related studies. The combination of these keywords aimed to capture the intersection of multi-material AM processes, optimization techniques, and the MMAM manufacturing method, providing a focused source for the literature review. Our literature search was structured using the PICO framework (Population, Interventions, Comparisons, Outcomes) as summarized in Table 1.

### 2.2. Paper Selection Process

Our selection process included two main stages:

Stage 1 comprised a review of material extrusion papers. The initial search returned 78 papers, which were then screened based on the following criteria:

- Relevance to construction: Priority was given to papers discussing applications, materials, or techniques with potential relevance or adaptability to additive manufacturing in architecture and the construction industry.
- Material relevance: Studies discussing materials with potential construction applications were prioritized.
- Optimization focus: Papers presenting novel optimization methods for multi-material AM were selected.
- Scalability potential: Studies demonstrating potential for scaling up to construction-sized applications were chosen.

Stage 2 consisted of the inclusion of papers from other AM methods. We broadened our scope to include papers relating to other AM techniques that provided valuable insights for construction applications, selected based on the following criteria:

- Innovative optimization methods: Papers presenting approaches that can be adapted to construction-scale material extrusion processes, such as those discussing machine learning or artificial intelligence approaches.
- Novel design approaches: Studies showcasing design strategies with potential architectural applications, including those discussing finite-element analysis or topology optimization.
- Multi-functionality: Research focused on optimizing multiple properties simultaneously.
- Validation techniques: Studies employing advanced testing methods applicable to large-scale AM components.

This two-stage process resulted in a final selection of 33 papers covering the intersection of multi-material AM optimization and potential construction applications.

### 2.3. Categorization and Analysis

The selected papers were systematically analyzed and categorized based on the following categories:

- Manufacturing processes.
- Material distribution and combination.
- Design approaches.
- Optimization methods.
- Validation methods.

**Figure 1:**
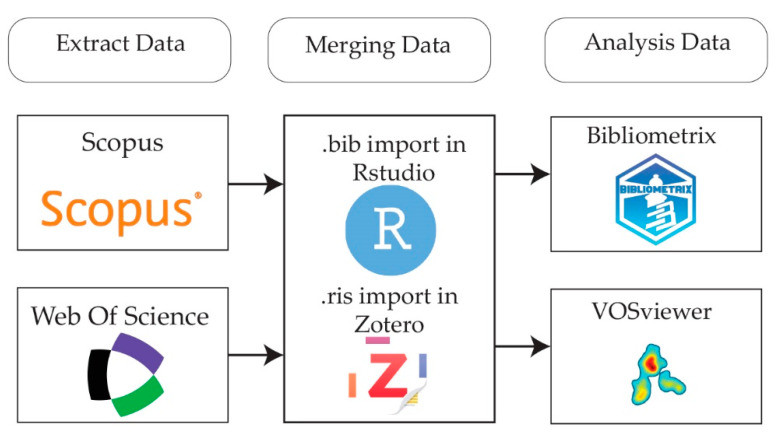
Data extraction, merging, and analysis workflow.

Data output was received from the Web of Science and Scopus scientific databases in (.bib) format. Since there are limitations on exporting more than 500 articles in Web of Science, the articles were sorted based on the most citations, and articles from number 1 to 500 were exported in the first export and the process continued until all papers were exported. Then, these files were merged using Notepad++ v8.6.7 software. The complete workflow for data extraction, merging, and analysis is illustrated in Figure 1.

**Figure 2:**
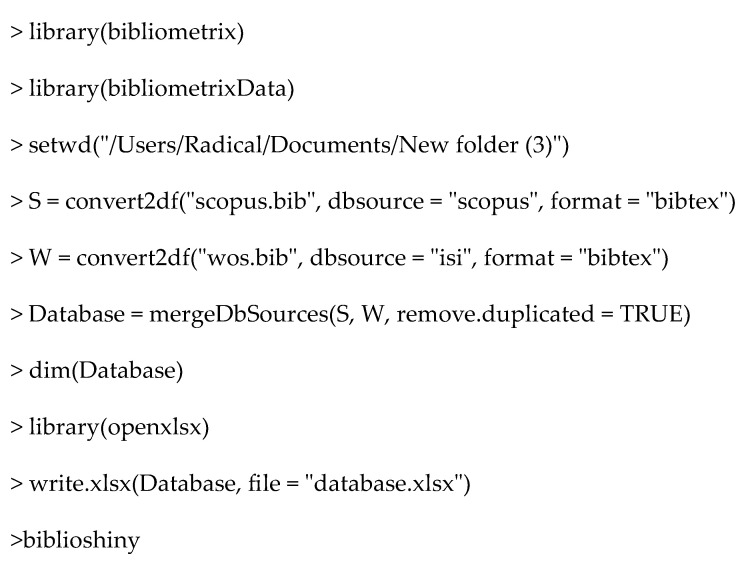
R code for bibliometric data processing and duplicate removal.

R and R Studio v2024.12.1 software was utilized. The commands used to merge data and remove duplicate data were as follows (Figure 2).

The methodology for this systematic review, including research questions, search strategies, and data synthesis approaches, is summarized in Table 2.

**Figure 3:**
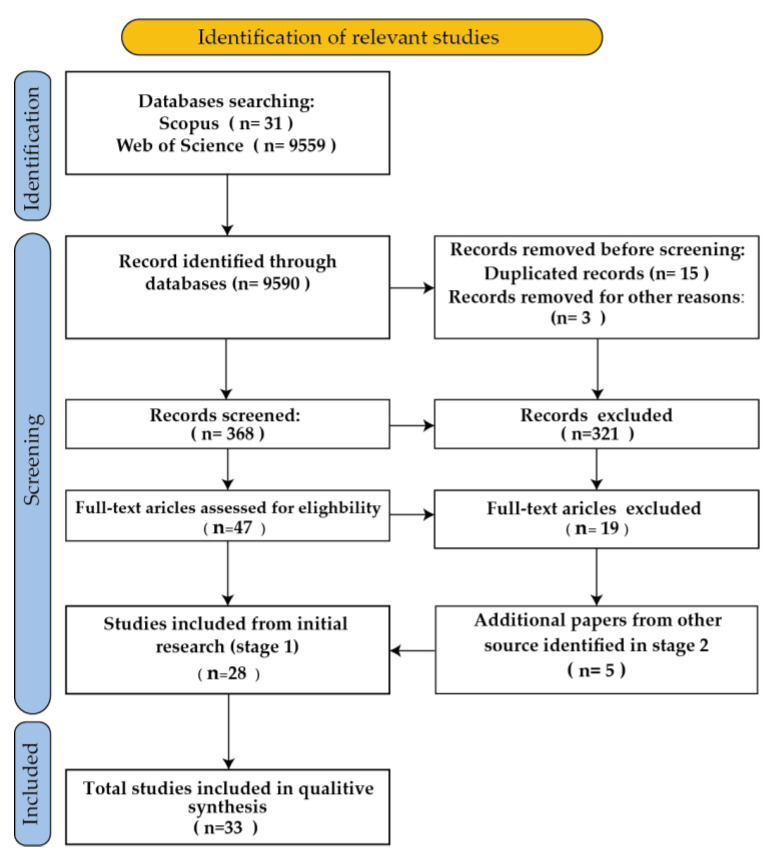
PRISMA diagram.

The PRISMA flow diagram (Figure 3) provides a visual representation of the literature search and selection process.

### 2.4. Bibliometric Analysis

**Figure 4:**
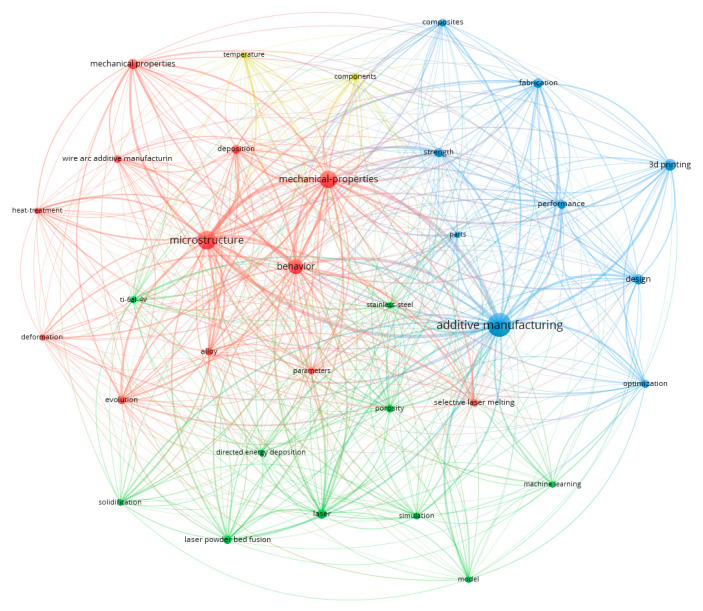
Additive manufacturing clusters.

The close and dense connection of clusters in the Vos-Viewer indicates the acceptance of additive manufacturing in the industry and the prevalence of the use of its new methods, as described in Figure 4.

**Figure 5:**
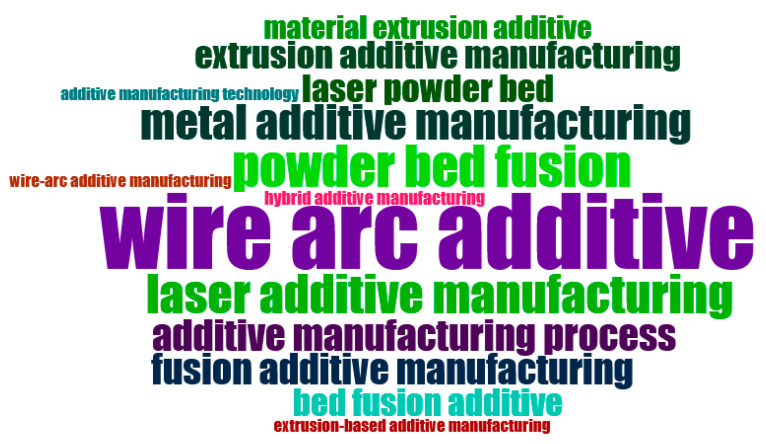
Word cloud image.

The word cloud (Figure 5) displays the terms that have been most utilized in this field of study. A larger word indicates a higher frequency of usage.

## 3. Key Parameters Influencing MMAM Processes

**Figure 6:**
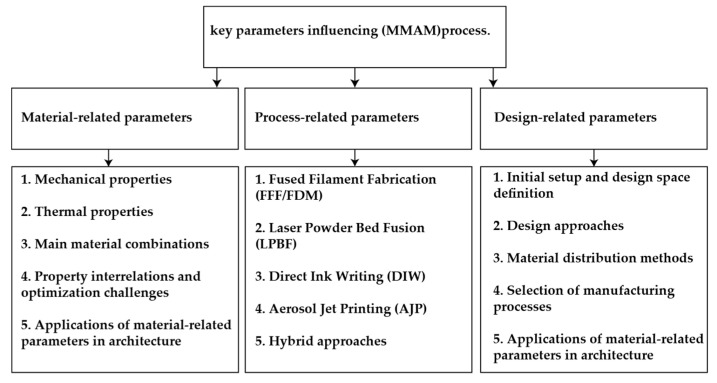
Overview of key parameters influencing MMAM processes.

Understanding the key parameters influencing multi-material additive manufacturing is essential for effective optimization and performance improvement. This section examines the critical factors, categorized into three main groups: (A) material-related, (B) design-related, and (C) process-related parameters. Each category will be examined in detail, drawing insights from diverse studies and applications. Figure 6 provides a visual overview of these parameter-based categories and their associated factors in this review study.

### 3.1. Material-Related Parameters

Mechanical, thermal, and functional properties, together with manufacturability and long-term performance, are all affected by material selection [10]. Therefore, the optimization of inter-material compatibility should be performed alongside process optimization to determine the required functions [4]. Considerations of material properties are essential for the optimization of multi-material additive manufacturing, since they affect both the manufacturing process and the performance of the product [10]. Table 3 summarizes key material parameters from the literature review, categorizing them into four main categories: mechanical properties; thermal properties; material combinations; and scale-dependent properties. Each category includes specific factors that significantly affect MMAM processes and final products.

#### 3.1.1. Mechanical Properties

The literature review reveals that MMAM enables the strategic gradation of key mechanical properties, including strength, stiffness, density, and ductility, within a single printed object. This distribution of properties allows designers to optimize structural performance by matching material characteristics to specific functional requirements in different regions of the component. Strength gradation enables localized performance optimization, as demonstrated by Wang et al.’s (2024) [11] core–shell structures, which achieved 30.1% improved damage absorption, while allowing strategic material placement that follows force trajectories through components [11]. Stiffness variation reduces stress concentrations at material interfaces by creating smooth transitions between regions with different elastic moduli, addressing a primary failure mechanism in traditional composites. Density control facilitates substantial weight reduction without compromising structural integrity, as exemplified by Herrmann and Sobek’s (2017) functionally graded concrete achieving up to 59% weight savings while maintaining load-bearing capacity. Enhanced ductility in specific regions improves overall toughness and crack resistance, which is particularly valuable in areas subject to tension or dynamic loading [12]. Together, these benefits enable levels of multi-criteria optimization that were previously unattainable with homogeneous materials [13].

Several proven methods exist for achieving mechanical properties gradation in MMAM. For strength variations, core–shell approaches utilize nested-material extrusion systems [11] or create selectively reinforced regions through fiber addition during printing. Stiffness gradients can be achieved through material blending in prescribed ratios, as demonstrated in Teawdeswan and Dong’s (2024) work with PLA/TPU combinations [14] and Suo et al.’s (2023) polymer blending guided by artificial neural networks [15]. Density control methods include varying infill percentages within single-material prints [16], and topology optimization algorithms that determine optimal material distribution [17]. Ductility enhancement typically involves selective fiber reinforcement, as in Niu et al.’s (2024) demonstration of effective carbon fiber placement in critical regions [18], or creating engineered interfaces between dissimilar materials, as shown in Englert et al.’s studies on mechanical interlocking structures [19]. These methods can be implemented individually or in combination, with computational design tools enabling multi-parameter optimization across various mechanical properties.

#### 3.1.2. Thermal Properties

In addition to its ability to vary mechanical properties, MMAM also enables the gradation of thermal properties. The primary thermal properties that can be graduated include thermal insulation, heat transmission, and heat dissipation. Insulation gradation enables the variation of thermal resistance across a component’s volume, as demonstrated in Han and Wei’s (2022) development of multi-material metamaterials with negative thermal expansion coefficients [20]. Heat transmission control facilitates the strategic channeling of thermal energy, creating designated pathways for heat flow that can direct or disperse thermal energy as needed, as illustrated by Xu et al.’s (2022) work on optimizing the electrothermal response of nozzle structures [21]. Enhanced heat dissipation in targeted regions allows for rapid temperature regulation where required, which is particularly valuable for components with integrated electronic or mechanical systems. Recent work by Ganguly and Tang (2025) demonstrates the potential of cellulose-based materials in MMAM, showing that cellulose acetate reinforced with cellulose nanocrystals achieved significant improvements in thermal stability (~28 °C increase in degradation temperature with 5 wt% CNCs) while creating lightweight, porous structures with enhanced mechanical properties through an innovative solvent-exchange post-processing technique [22]. As shown in the studies, these thermal gradations can function simultaneously with mechanical property optimizations, creating multi-functional components that address both structural and thermal requirements.

Several methods have been developed to achieve the gradation of thermal properties in MMAM processes. Multi-material structures employing controlled transitions between materials with contrasting thermal conductivity enable the development of fine-tuned insulation gradients throughout a single component, as seen in Quero et al.’s (2022) work integrating PETG with conductive materials for microfluidic devices [23]. Xu et al.’s (2022) optimization of electrothermal response through fuzzy adaptive control offers insights into methodologies for controlling heat transmission through tailored material distribution [21]. As for heat dissipation, internal geometries can create varying surface area-to-volume ratios within different regions, enhancing cooling efficiency where needed [17]. These methods can be further enhanced through topology optimization algorithms specifically focused on thermal properties, in like manner to the approaches used for mechanical property optimization.

#### 3.1.3. Scale-Dependent Properties

Scale-dependent properties are material characteristics that exhibit different behaviors or values depending on the dimensional scale at which they are observed or measured [24]. In MMAM, these properties are categorized across three scales: microscale (≤1 mm), in which interfacial phenomena predominate, including surface tension, as well as adhesion strength between dissimilar materials, as demonstrated by Englert et al [19]. through their mechanical interlocking structures; mesoscale (1 mm to 10 cm), which bridges microscopic and macroscopic behaviors through cellular structures, with key properties including effective elastic moduli and relative density, as worked out by Teawdeswan and Dong [14] in their gyroid structures; and macroscale (>10 cm), in which global mechanical properties emerge, such as structural stiffness, thermal expansion, and time-dependent behaviors, along with failure mechanisms including delamination, warping, and buckling, which become dominant concerns at these larger dimensions, as identified by [13] in their large-scale fabrication studies. Bartkowiak et al. further demonstrated how surface wettability exhibits scale-dependent behavior, with the strongest functional correlations occurring at specific microscale dimensions (~1100 μm^2^), revealing interactions that conventional characterization methods might overlook [25].

#### 3.1.4. Main Material Combinations

**Figure 7:**
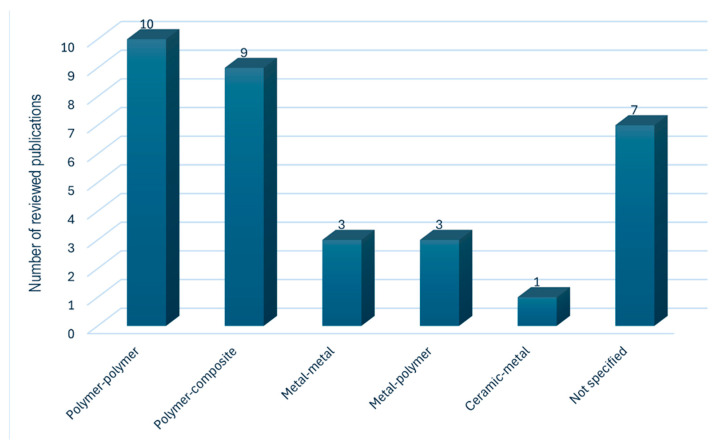
Material combinations in MMAM processes.

This study has identified several material combinations used in MMAM, each presenting unique optimization challenges. As shown in Figure 7, polymer-based materials are the most common, with polymer–polymer and polymer–composite combinations being the most employed.

Polymer-based combinations are favored for their adaptability and compatibility with fused filament fabrication (FFF) methods, but require process parameter optimization to balance properties such as strength and flexibility [14,15,16,17,18,19,20,21,22,23,24,25]. Metal–metal and metal–polymer combinations pose more sophisticated optimizing challenges, often necessitating multi-objective approaches to balancing mechanical properties, printability, and interfacial bonding [26,27].

Notably, some papers in this review, for example those of Xu et al., Zhang et al., and Ceclic et al., did not specify particular combinations of materials but rather concentrated on other factors affecting multi-material additive manufacturing [21,28,29].

#### 3.1.5. Property Interrelations and Optimization Challenges

The properties, combinations, and scale-dependent characteristics of materials in MMAM are often interconnected, creating complex optimization constraints. These relationships include mechanical, thermal, and interfacial aspects, defining several variables to consider during the production process [18,30]. For example,

- Enhancing tensile strength might require changes in printing temperatures, subsequently impacting rheological behavior and thermal gradients within the printed parts [31];
- Managing differences in thermal expansion between materials requires trade-offs with processing temperatures or print speeds [32];
- The optimization of the bonding between metals and polymers may require changing processing parameters, which then influences the resulting microscale structure and macroscale properties [19,27];
- Adjustments made to achieve the required mechanical properties can impact thermal performance, and vice versa, demanding careful evaluation of both parameters to ensure optimal results [33,34].

These interrelated factors form a complex optimization landscape, in which improving one aspect can lead to compromises in another. Consequently, MMAM processes frequently require advanced methods for parameter selection and material combination to achieve the required balance of properties in the final product. Current research is centered on developing precise predictive models for material interactions, optimizing interfacial bonding between dissimilar materials, and creating comprehensive material databases suitable for MMAM processes [14,15,28].

#### 3.1.6. Applications of Material-Related Parameters in Architecture

The applications of material-related parameters in multi-material 3D printing have the potential to advance building practices, offering unique opportunities for high-performance, sustainable architectural solutions. One of the most significant opportunities lies in optimizing mechanical properties to create structurally efficient building elements [35]. As demonstrated in the works of Liu et al. and Khan et al. [16,31], MMAM technology can precisely combine materials with complementary properties, as seen in tensile multi-material specimens. In architecture, Craveiro et al.’s work on 3D printing functionally graded concrete materials has already shown that MMAM can be applied to construction materials [35]. Building on these advancements, this review identifies specific areas of MMAM application that could enhance architectural design and construction techniques.

Thermal property control through MMAM offers another avenue for architectural innovation. Brailovski et al.’s work on controlling material properties in laser powder bed fusion processes suggests possibilities for creating components with tailored thermal characteristics [33]. In architecture, this could be applied to develop energy-efficient building envelopes with customized insulation properties, significantly reducing heating and cooling demands and contributing to more sustainable building operations. Projects like Duballet et al.’s work on graded polystyrene aggregate concrete structures have explored thermal performance in construction [36].

Craveiro et al.’s research on the optimization of building components, using voxels as a method for the development of multi-material elements, has dealt with the process of designing combinational materials based on voxel evaluation [35]. The formulation of construction parts with varying degrees of material stiffness is considered an effective strategy for optimizing several criteria [37]. Our review suggests the potential for achieving precisely controlled thermal gradients across entire building envelopes. The exploration of triply periodic minimal surface (TPMS) structures by Chaffins et al. suggests the potential for the creation of components with spatially varying properties [38]. Such methods can be applied to flooring systems with tailored acoustic properties for optimal sound control in different areas of a building. These potential applications of MMAM in architecture closely align with findings from our literature review. This technology has the potential to enable the meeting of several performance-based criteria simultaneously, optimizing resources use, and addressing complex high-performance building needs.

### 3.2. Process-Related Parameters in Multi-Material Additive Manufacturing

**Figure 8:**
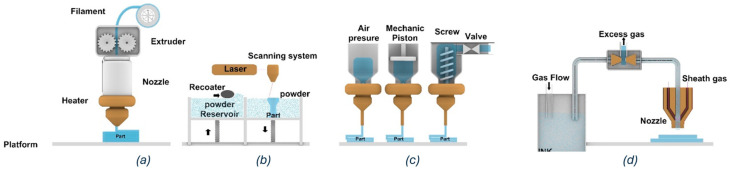
Schematic representations of key MMAM processes: (**a**) fused filament fabrication (FFF), (**b**) laser powder bed fusion (LPBF), (**c**) direct ink writing (DIW), and (**d**) aerosol jet printing (AJP).

Building upon the material-related parameters discussed earlier, this section examines the critical process parameters across four MMAM techniques: fused filament fabrication (FFF), laser powder bed fusion (LPBF), direct ink writing (DIW), and aerosol jet printing (AJP). Notably, in relation to our research focus, FFF emerges as the dominant fabrication method. Figure 8 provides schematic representations of these key MMAM processes, illustrating their fundamental operating principles.

**Figure 9:**
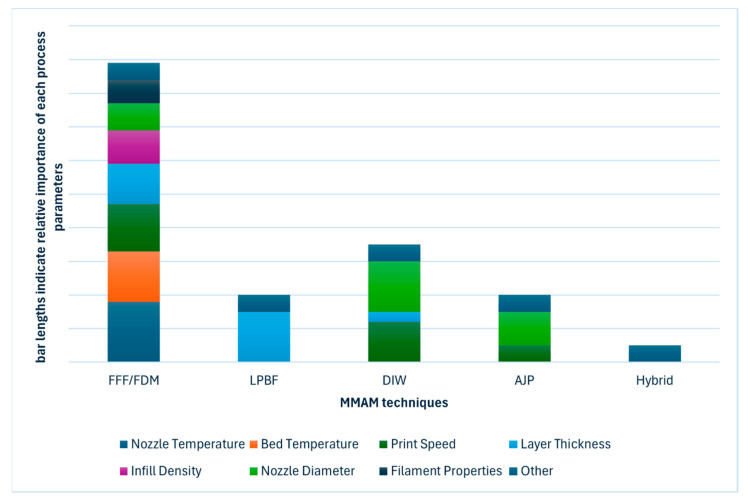
Relative importance of process parameters in MMAM techniques.

Each of these techniques has specific process parameters that influence the structural integrity, dimensional accuracy, layer adhesion, functionality, and mechanical performance of the manufactured parts. Figure 9 illustrates the relative importance of these parameters; these values were quantified based on the frequency of parameter citations across publications. As shown in Figure 9, each MMAM technique has a distinct set of parameters, with FFF/FDM showing a particularly complex interplay of factors. This section examines these parameters in detail, highlighting their impact on both material properties and functional performance.

#### 3.2.1. Fused Filament Fabrication (FFF/FDM)

As illustrated in the FFF schematic in Figure 8a and the parameter importance shown in Figure 9, nozzle temperature, printing bed temperature, and print speed are the most critical parameters for this process. Recent studies support this finding: Chen et al. demonstrated that nozzle temperature affects the tensile strength of PETG/ABS bi-layer specimens [39], while Khan et al. emphasized the role of bed temperature in preventing warpage and ensuring proper first-layer adhesion in multi-material prints [31]. 

#### 3.2.2. Laser Powder Bed Fusion (LPBF)

In LPBF, laser power and scan speed are dominant factors. Brailovski et al. demonstrated their effects on part density and grain structure in Ti-18Zr-14Nb [33]. Wang et al. further explored how these parameters, along with hatch spacing, influence the thermal stress and fluid dynamics during the process [26].

#### 3.2.3. Direct Ink Writing (DIW)

In DIW, ink viscosity and extrusion pressure are primary concerns. Quero et al. showed the importance of these concerns in achieving high-resolution features in multi-material microfluidic devices [23]. Alsharif et al. further investigated how these parameters, combined with nozzle diameter and print speed, affect the quality of printed parts [40].

#### 3.2.4. Aerosol Jet Printing (AJP)

In AJP, the carrier gas flow and sheath gas flow are critical parameters. zhang et al. conducted a comprehensive study covering multiple AJP parameters, emphasizing the importance of maintaining the delicate balance involved in achieving high-quality deposition in fine-feature applications [28].

#### 3.2.5. Hybrid Approaches

Hybrid MMAM techniques present unique challenges. Englert et al. investigated LPBF-FFF integration, focusing on temperature control and multi-material interfaces [19]. Niu et al. explored a combination of material extrusion and laser processes, addressing issues related to process organization and material transition issues [18]. It is important to note that most studies simultaneously consider multiple parameters, recognizing the complex interactions between them. For instance, Chen et al. examined nozzle temperature, bed temperature, and print speed [39], while Brailovski et al. considered laser power, scan speed, and layer thickness together [33].

### 3.3. Design-Related Parameters

Design-related parameters connect material capabilities and manufacturing constraints, shaping the functionality of MMAM-produced components. This section shows how design choices both influence and are influenced by the material properties and process parameters discussed above. We will explore how designers navigate the complex landscape of MMAM, balancing multiple goals while adhering to various constraints. Our analysis will cover the main aspects of MMAM design, including the following:

- Initial setup and design space definition;
- Design approaches;
- Material distribution methods;
- The choice of manufacturing process;
- Design validation and characterization.

**Figure 10:**
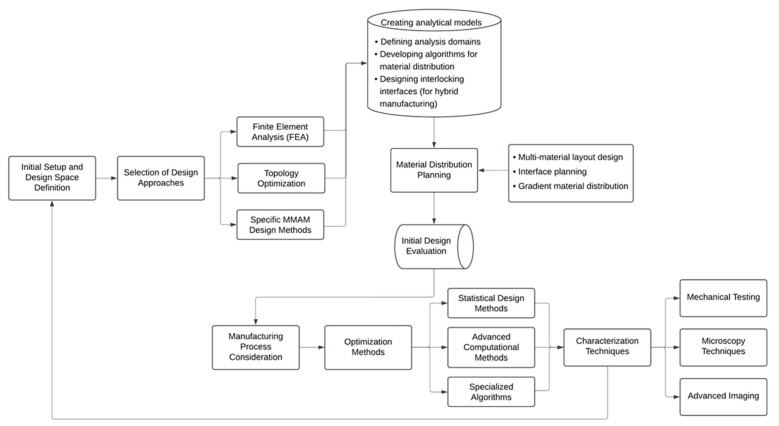
Key steps and process parameters in MMAM techniques.

Figure 10 outlines the key steps and relationships in the MMAM design process, providing a visual reference for our subsequent discussion. As we explore each aspect, we will draw connections to the optimization techniques and objectives discussed in other sections of this paper.

Table 4 summarizes the various approaches researchers have taken in their work, including those related to initial setup methods, design methodology, manufacturing process, optimization techniques, and characterization methods. These approaches address various challenges associated with multi-material additive manufacturing. As shown in Table 4, researchers are using diverse design workflows to tackle the unique challenges of MMAM. These approaches range from topology optimization for specific structures, such as auxetic structures and non-pneumatic tires, to algorithms for multi-material distribution and interface design. The table shows the interdependence of design parameters with manufacturing processes and material selection, underscoring the complexity of MMAM design optimization.

#### 3.3.1. Initial Setup and Design Space Definition

Defining the problem scope, constraints, and objectives is fundamental to any design and optimization process in MMAM. Recent studies highlight the importance of tailoring the initial setup to specific applications and design goals. For instance, Giubilini and Minetola utilized a re-entrant auxetic structure model for automotive suspension components through analytical modelling, exploring the potential of multi-material printing to create functional deformable structures [41]. In contrast, Liu et al. focused on domain analysis by developing an analysis domain for a compliant-finger mechanism to facilitate multi-material design optimization [16]. Dezianian and Azadi used MATLAB as a computational tool to address the non-pneumatic tire design problem, demonstrating how computational tools can effectively integrate multiple objectives within the design space [42]. These varied approaches stress the need for flexibility when initializing MMAM designs to meet various application requirements.

#### 3.3.2. Design Approaches

Advancements in MMAM have led to the development of various design techniques to address complicated manufacturing problems associated with multifunctional components [10,43]. This review shows that these methodologies are often used in combination. Table 5 classifies these strategies into eight major categories. The table highlights key elements of each design approach, including an overview, the associated process, and examples from reviewed publications.

**Figure 11:**
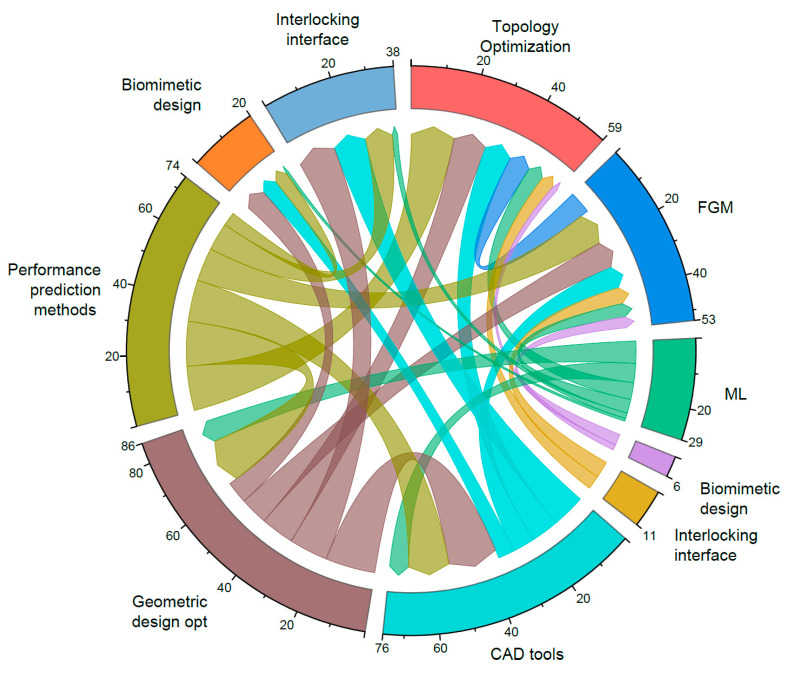
Chord diagram illustrating the interconnections between various design approaches.

To visualize the connections between these design approaches, the collective use of different design methods in individual studies was systematically recorded and quantified. These data were then used to create a co-occurrence matrix. This matrix formed the basis for generating a chord diagram (Figure 11) which visually represents the frequency and strength of the relationships between the different methods.

Figure 11 shows that performance prediction methods are strongly connected to other approaches, particularly with topology optimization and geometric design optimization methods. CAD modeling and geometric design optimization also exhibit strong associations with most other methods, reflecting their widespread use in many ways in MMAM design processes. Functionally graded material design shows moderate connections across various methods, indicating its versatility. Machine learning-aided strategies, while less prevalent, still have notable links to performance prediction and biomimetic design. The interlocking interface is particularly linked to geometric design optimization and CAD modeling, emphasizing its importance in integrating multi-material components. As the field evolves, we can expect greater integration between these methods and the emergence of advanced design strategies.

#### 3.3.3. Material Distribution Methods

From the works in the literature, we have identified four primary distribution approaches: discrete, gradient, hybrid, and temporal (4D printing). Each aligns with specific design strategies from the eight major categories outlined in the previous section. Table 6 summarizes these primary material distribution methods in MMAM, highlighting key design considerations, schematic representations of material distribution patterns, and examples from recent research.

The selection of distribution methods often overlaps with various design approaches. For example, the interlocking interface design studies by Englert et al., which used different interlocking geometries in metal–polymer hybrid components, variously employ discrete and hybrid distribution methods [19]. Similarly, CAD modeling supports the application of all distribution methods, allowing designers to precisely control material placement. Machine learning approaches, as demonstrated by Zhang et al. and Wang et al., can be applied to optimize any of these distribution methods [28,30]. In another example, Teawdeswan and Dong used neural networks to predict design parameters for gyroid structures with different material ratios, possibly leading to novel hybrid strategies that blur the lines between distribution types [14].

#### 3.3.4. Selection of Manufacturing Processes

A review of the literature reveals that each printing technology has distinct capabilities and challenges related to material placement strategies and design tasks. Table 7 provides an overview of the main MMAM processes, outlining their strengths and limitations, and referencing relevant studies from our review.

Fused filament fabrication (FFF) stands out as the process most extensively explored in the reviewed literature. It has been shown to support both discrete and gradient material distribution. Research by Chen et al. [39] and Khan et al. [31] demonstrated its effectiveness in achieving discrete distribution patterns and intricate cross-sectional materials. However, Wang et al. [30] pointed out that FFF faces challenges such as anisotropic properties and limited interface strength between materials.

Laser powder bed fusion (LPBF) has been explored in the context of producing components with gradient material distributions in metals. Brailovski et al. [33] developed general processing maps for LPBF to adjust the properties of intermediate materials. While LPBF is known for its ability to achieve high density and strength in components, many researchers are careful to point out inherent challenges such as residual stress and complex thermal management; see, for example, Wang et al. [26].

Direct ink writing (DIW) and aerosol jet printing (AJP) are particularly useful for applications requiring high precision. Quero et al. [23] showed that DIW is effective in designing high-resolution micro-structured fluid carriers, with a focus on applications relevant to biomimicry. Alsharif et al. [40] further expanded DIW’s potential by producing stretchable multi-material electrodes, broadening the functional applications of the method. Yet, DIW is limited to specific material viscosities, and careful optimization of printing parameters is crucial to obtaining the desired results. Zhang et al. [28] demonstrated AJP’s capacity to precisely control material distribution through AI-driven optimization, particularly in complex structures like electronics and sensors. While AJP provides a solution for material deposition, it is limited by challenges in process parameter sequencing and scalability. To fully harness AJP’s potential, advanced control mechanisms are necessary.

These studies highlight the growing importance of innovative manufacturing technologies in overcoming the challenges of achieving geometrical and functional complexities in MMAM. Nevertheless, a substantial gap remains, particularly in the comparison of these processes, and especially in terms of the characteristics and quality of the final parts. Future work should focus on developing a more structured methodology for process selection in MMAM, exploring the integration of various techniques to leverage their combined strengths, and advancing the monitoring and control systems used during processing.

## 4. Optimization Techniques in MMAM

**Figure 12:**
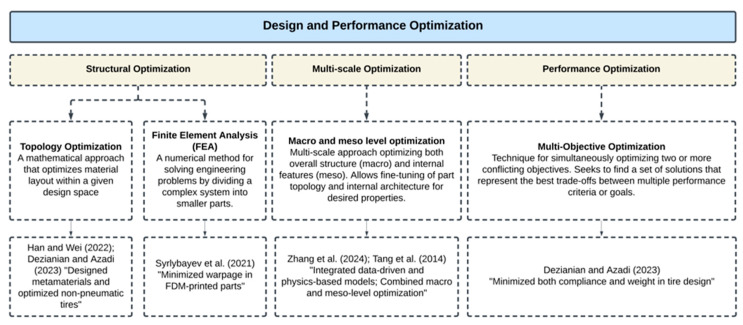
Optimization strategies for design and performance in MMAM.

**Figure 13:**
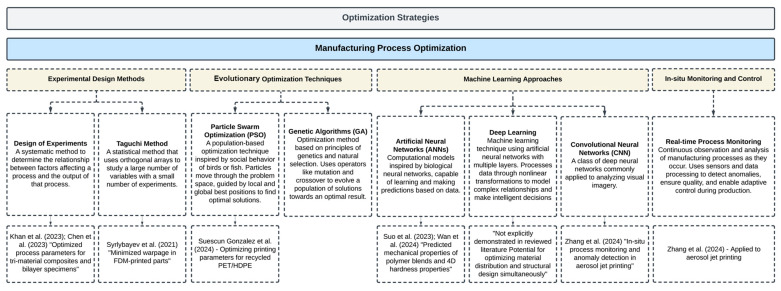
Manufacturing process optimization strategies in MMAM.

Figure 12 and Figure 13 offer an overview of the optimization techniques in MMAM, summarizing the main strategies, along with brief descriptions and lists of related studies. These figures show a roadmap for the discussion that follows, illustrating the breadth and diversity of optimization approaches highlighted in the MMAM studies reviewed in this paper.

### 4.1. Empirical and Statistical Methods

Empirical and statistical methods provide essential frameworks for optimizing MMAM processes. Techniques such as design of experiments (DOE) and response surface methodology (RSM) have proven effective in analyzing complex parameter interactions across diverse MMAM processes. Additionally, the Taguchi method offers an efficient strategy for resolving quality-related issues, making it particularly useful for addressing specific manufacturing challenges in MMAM [31,39,43].

### 4.2. Computational Methods

Computational techniques offer predictive tools for optimizing MMAM processes. finite-element analysis (FEA) plays a critical role in both design and process optimization, especially in overcoming challenges related to multi-material interfaces [47]. Multiphysics modeling, crucial for processes involving multiple physical considerations, enables comprehensive simulation of complex MMAM processes [48]. While these methods offer significant insights, they often require substantial computational resources and experimental validation.

### 4.3. Advanced AI/ML Approaches

The integration of artificial intelligence (AI) and machine learning (ML) represents a significant advancement in MMAM optimization [49,50]. Artificial neural networks (ANNs) and convolutional neural networks (CNNs) have shown promise in predicting material properties, optimizing process parameters, and enabling real-time process monitoring [18,51]. Fuzzy adaptive control methods also offer novel approaches to handling uncertainty in MMAM processes [20]. However, despite their potential, AI/ML approaches face challenges in data acquisition, model interpretability, and generalization across diverse MMAM applications.

### 4.4. Multi-Objective Optimization

The multi-faceted nature of MMAM often necessitates multi-objective optimization approaches. Pareto optimization and weighted-sum methods have emerged as key strategies for balancing competing objectives in MMAM design, allowing for the exploration of trade-offs between various performance criteria [16,42,45].

## 5. Design Validation and Characterization

The final important step in any AM process involves validating and characterizing the manufactured parts to ensure they meet specifications and perform as expected in real-world scenarios [52]. Our review reveals a diverse array of methods employed by researchers to thoroughly assess MMAM components. Table 8 provides a comprehensive overview of these validation and characterization methods across the various MMAM processes discussed in this review.

As shown in Table 8, validation and characterization methods can be subdivided into the following two broad categories: physical and mechanical characterization, and functional and application tests. Physical and mechanical characterization methods are basic tools for functional design verification of MMAM components [54]. These methods are common in investigations relating to fused filament fabrication (FFF) [55] and laser powder bed fusion (LPBF). For example, Khan et al. and Chen et al. conducted tension tests in evaluating the strength of interfaces in multi-material FFF parts [31,39]. Similarly, in the context of LPBF, Brailovski et al. reported on compression tests on parts manufactured using a Ti-18Zr-14Nb alloy [33]. Internal structural analysis of printed parts involves the use of techniques such as scanning electron microscopy (SEM), X-ray diffraction (XRD), optical microscopy, and other microstructural analysis technologies [18]. Huang et al. combined these techniques in their study of LPBF Ti6Al4V/AlMgScZr-based multi-materials [34]. Functional and application-specific testing methods are typically designed to evaluate properties produced by manufacturing techniques. For instance, Wu et al. performed pH responsiveness tests on 4D printed structures made by a custom FFF process [46]. In microfluidics, Quero carried out electrical resistance tests on devices fabricated using conductive filaments via FFF [23]. Zhang et al. and Wang et al. used the results of functional tests to refine design characteristics and optimize manufacturing parameters, thereby ‘closing the loop’ to enhance the final product [28,30].

### Applying Multi-Material Additive Manufacturing

Our review acknowledges progress in multi-material additive manufacturing (MMAM) for architecture and construction, as shown by Pajonk et al. and Vargas et al. [2,37]. However, we see opportunities to advance and implement these technologies further. Based on our review of current research and trends, we propose ways to advance MMAM in architectural applications.

Table 9 highlights five key areas where significant improvements can be made: Equipment Design, Process Control, Design Integration, Digital Tools, and Interdisciplinary Integration. For each area, we compare current methods with proposed improvements based on emerging research and technological capabilities.

## 6. Discussion

The current design tools, for architectural MMAM, have significant limitations. Traditional CAD programs lack specialized capabilities for multi-material design and optimization. The chord diagram in Figure 11 highlights the interdependencies of design approaches and reveals the underdevelopment of integrated workflows connecting these methods. While topology optimization has been effective in engineering applications [20,42], its adaptation to architectural scales remains limited. Dezianian and Azadi applied topology optimization to multi-material structures, successfully minimizing compliance under weight constraints [42]. However, scaling these methods for architecture requires addressing distinct loading conditions, material constraints, and manufacturing limitations. AI and machine learning offer promising avenues for MMAM optimization in architecture by enabling multi-criteria performance optimization across scales. However, their effectiveness depends on the availability of extensive datasets tailored to architectural materials and performance requirements.

**Figure 14:**
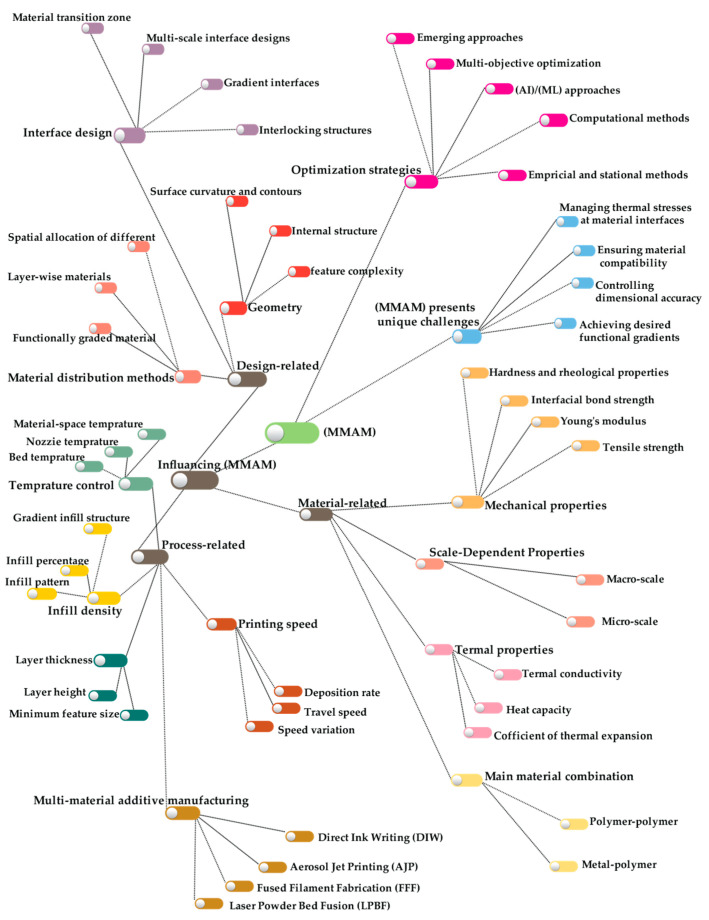
Overview of the interconnected factors in MMAM.

A significant gap concerns the relationship between material-scale properties and building-scale performance. While studies like [15] demonstrate control over specific material properties like negative thermal expansion and negative Poisson’s ratio in metamaterials, translating these capabilities to meaningful building performance improvements requires multi-scale modeling approaches. The challenge lies in connecting microscale material design with macroscale architectural performance. Most of the existing research efforts have used empirical methodologies without comprehensive finite-element analysis validation. While some studies, like [24], employed FEA methods to predict deformation in composite structures, showing good agreement with experimental results for certain geometries, most of the research lacks the multiphysics-based simulation necessary for architectural applications. This becomes particularly problematic for architectural applications in which safety factors and performance guarantees are essential regulatory requirements. The minimal focus on software development specifically for architectural MMAM represents a significant implementation barrier. Practical architectural applications of MMAM require solving these challenges spanning materials science, manufacturing, and design. Figure 14 provides an overview of the multiple factors and relationships in MMAM, illustrating the complex interdependencies between design-related, material-related, and process-related parameters.

## 7. Conclusions

**Figure 15:**
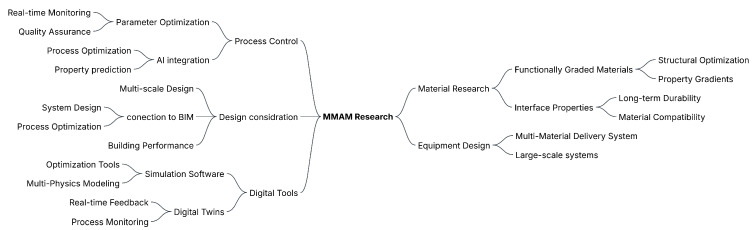
Mind map for future MMAM research in architecture and construction.

This review has examined the various aspects of design optimization and process parameter control in multi-material additive manufacturing (MMAM), with a particular focus on implications for architecture and construction. Figure 15 provides a detailed overview of the interconnected factors influencing MMAM processes and outcomes.

The synthesis of recent studies has revealed several key findings and their implications for future research in construction industry, as detailed in Table 10.

Building on our review findings, we present a framework of five key domains for advancing MMAM in architecture: Equipment Design, Process Control, Design Integration, Digital Tools, and Materials Research. These domains cover both current capabilities and future development needs in building construction-scale MMAM applications.

Progress in these domains is deeply interconnected. Advances in Equipment Design affect Process Control capabilities, while Design Integration relies on effective Digital Tools. Materials Research forms the foundation for all other domains, determining both manufacturing limits and performance outcomes. This framework serves as a road map for future research.

## Figures and Tables

**Table 1:** PICO table.

No.	Item	Context
1	Population	Optimization techniques in MMAM in the building industry.
2	Interventions	All instances referring to the status of AM and MMAM.
3	Comparisons	FGM researchers, process and information technologies.
4	Outcomes	The role of AM in industry’s use of MMAM.
5	Study method	Quantitative and qualitative analysis. ^1^

^1^ A systematic Boolean search.

**Table 2:** Research protocol.

No.	Item	Content
**1**	Aim	
	Key Objective	To establish contextual knowledge relating to AM- MMAM through the analysis and classification of articles.
**2**	Research Questions	
	Main Question	Is there a need for a comprehensive optimization framework, the integration of data-driven approaches, and real-time monitoring in MMAM?
	Sub-questions	How can new construction technologies be introduced to industry? What are the optimized integration methods for AM and MMAM?
**3**	Search Methods	
	Electronic Databases	Scopus; Web of Science
**4**	Selection	
	Scopus String	(TITLE-ABS-KEY (“Additive manufacturing”) OR TITLE-ABS-KEY (“rapid prototyping”) AND TITLE-ABS-KEY (“Functionally graded materials”) OR TITLE-ABS-KEY (“Direct digital manufacturing”) AND TITLE-ABS-KEY (“Functionally graded materials”) OR TITLE-ABS-KEY (“gradient materials”) OR TITLE-ABS-KEY (“multi material”) OR TITLE-ABS-KEY (“Process optimization”) OR TITLE-ABS-KEY (“function optimization”) OR TITLE-ABS-KEY (“functional optimization”) OR TITLE-ABS-KEY (“performance optimization”) OR TITLE-ABS-KEY (“mechanical optimization”) OR TITLE-ABS-KEY (“thermal optimization”) OR TITLE-ABS-KEY (“optimization”) AND TITLE-ABS-KEY (“Fused deposition modeling”) OR TITLE-ABS-KEY (“fused filament fabrication”) OR TITLE-ABS-KEY (“material extrusion”) OR TITLE-ABS-KEY (“layer extrusion printing”)) AND PUBYEAR > 2019 AND PUBYEAR < 2026
	Web of Science String	“ADDITIVE MANUFACTURING” (Title) OR “rapid prototyping” (Title) OR “direct digital manufacturing” (Title) AND “functionally graded materials” (Title) OR “gradient materials” (Title) AND “multi material” (Title) OR “multiple materials” (Title) AND “process optimization” (Title) OR “function optimization” (Title) AND “functional optimization” (Title) AND “performance optimization” (Title) OR “mechanical optimization” (Title) OR “thermal optimization” (Title) OR “optimization” (Title) AND “fused filament fabrication” (Title) OR “material extrusion” (Title) OR “layer extrusion printing” (Title) and 2021 or 2022 or 2023 or 2024 or 2025 (Publication Years) and Article (Document Types)
	Idiom	English
**5**	Exclusion criteria	
		Not related to AMMM or AM.
		Abstract not available for download.
		Not written in the defined idiom.
**6**	Search Method	
	Data Extraction	Use R software to preserve the integrity of data and monitor status based on the protocol.
	Narrative Synthesis	Bibliometric analysis: Publications over time; Methodology applied. Thematic analysis: Analysis of selected publications; Description of findings, outcomes and relationships.

**Table 3:** Key material properties influencing MMAM processes.

Property Type	Property/Combination	Influence on MMAM	Key Study
Mechanical	Young’s modulus	Affects part stiffness and deformation	[5]
Mechanical	Tensile strength	Influences load-bearing capacity and durability	[6]
Mechanical	Flexural properties	Impacts bending behavior and structural integrity	[7]
Mechanical	Hardness	Affects wear resistance and surface quality	[8]
Mechanical	Rheological properties	Influences printability, layer adhesion, and part quality	[8]
Mechanical	Interfacial bonding	Affects part integrity and performance	[9]
Thermal	Thermal conductivity	Affects heat distribution and dissipation during printing	[10]
Thermal	Thermal expansion coefficient	Critical for managing residual stresses and warping	[4]
Thermal	Melting point	Affects material behavior during printing	[11]
Thermal	Glass transition temperature	Impacts material flow and layer adhesion	[11]
Material Combinations	Polymer–polymer	Balances properties like strength and flexibility	[11]
Material Combinations	Metal–metal	Optimizes mechanical properties and processability	[10]
Material Combinations	Polymer–composite	Enhances base polymer properties	[9]
Material Combinations	Metal–polymer	Influences material behavior at molecular/microscopic level	[12]
Scale-Dependent	Micro-scale properties	Influences material behavior at molecular/microscopic level	[13]
Scale-Dependent	Macro-scale properties	Determines bulk properties observable at macroscopic level	[6]

**Table 4:** Summary of design workflows for MMAM.

Study	Initial Setup/ Design Space	Design Approach	Manufacturing Process	Optimization/ Design Process	Characterization Method
Giubilini and Minetola [41]	The analytical model of a reentrant auxetic structure.	Topology optimization for auxetic structures	Fused Filament Fabrication (FFF)	-	Dimensional analysis using CT scan
Dezianian and Azadi [42]	Problem defined in MATLAB code.	Topology optimization for non-pneumatic tire	Laser Powder Bed Fusion (LPBF)	Optimization steps	Compression tests
Liu et al. [16]	Analysis domain of the compliant finger.	Multi-material topology optimization for compliant mechanisms	Fused Filament Fabrication (FFF)	Topology optimization results for single-material and bi-material designs	Tensile tests
Englert et al. [19]	Setup for manufacturing hybrid LPBF-FFF specimens.	Design of interlocking interfaces for hybrid manufacturing	Hybrid LPBF–FFF	Various interlocking geometries and their fabrication paths	Tensile tests, shear tests, and μCT analysis
Suescun Gonzalez et al. [13]	Global framework of the study.	Direct recycling and printing of multi-material waste	Fused Granular Fabrication (FGF)	-	FTIR, DSC, MFI, density measurements, and tensile tests
Han and Wei [20]	Principle for introducing numerical homogenization method	Alternating Active Phase and Objective (AAPO) algorithm for multi-material topology optimization	Multi-material Fused Deposition Modeling (FDM)	Topology optimization results for different parameter settings	Poisson’s ratio and thermal expansion experiments, FEA verification
Wang et al. [11]	Schematic diagrams of component proportions.	Machine learning prediction of hardness for multi-material blends	Fused Filament Fabrication (FFF) with custom mixing nozzle	Machine learning model optimization and comparison	Shore hardness measurements, machine learning prediction validation
Khan et al. [31]	FFF 3D printer and tensile test samples.	Full factorial design of experiments, ANOVA analysis	Fused Filament Fabrication (FFF)	Optimization of FFF processing parameters.	Tensile testing, SEM, and optical microscopy
Xu et al. [21]	Heat structures including heating and radiation, and assembly fastening form in RP.	Electrothermal response optimization of nozzle structure for multi-material RP based on fuzzy adaptive control	Fused Deposition Modeling (FDM)	(a) Iterative optimal value of C and calculation of RSS; (b) different α and corresponding minimum RSS	Thermal–solid coupling finite-element analysis, physical experiments measuring temperature change and material extrusion loading

**Table 5:** Classification of design methods for MMAM.

Design Methods	Description	Methodology	Example References
Topology optimization	Method to optimize material distribution within a given design space for improved performance while satisfying given constraints [4].	Uses algorithms to iteratively remove or redistribute material based on stress analysis, often employing finite-element analysis (FEA) to evaluate designs [5,6,38].	Liu et al. [16], Han and Wei [20], Dezianian and Azadi [42].
Functionally graded material design	Creating components with spatially varying composition or structure for specific performance gradients [3].	Involves designing continuous or stepwise variations in material composition or properties across a part, often using parametric modeling or optimization algorithms [15].	Han and Wei [20].
Biomimetic design	Employing engineering solutions based on nature’s structures and processes to solve technical problems [13].	Analyzes and mimics structures and mechanisms found in nature, often involving reverse engineering of biological systems and adapting them to engineering problems [24].	Wang et al. [11].
Interlocking interface design	Creating companion geometries at material interfaces to enhance bond strength and component integrity in multi-material structures [11].	Designs specific geometric features at material interfaces to improve mechanical interlocking, often using CAD modeling and FEA to optimize interface geometry [11].	Englert et al. [19], Cecil et al. [29], Teawdeswan and Dong [14].
Geometric design optimization	Optimizing part geometry to improve performance or meet specified criteria, often focusing on specific features or overall shape [40].	Uses parametric design and optimization algorithms to adjust geometric features, often in conjunction with FEA or other performance simulation methods [40].	Giubilini and Minetola [41], Alsharif et al. [40], Zhang et al. [28].
CAD modeling	Using computer-aided design software to create and modify complex MMAM designs, enabling precise control over part geometry and material distribution [27].	Employs 3D modeling software to create digital representations of parts, often incorporating parametric design features for easy modification and optimization [18,27].	Quero et al. [23], Cecil et al. [29].
Performance prediction methods	Utilizes methods like finite-element analysis (FEA) and computational fluid dynamics (CFD) to simulate part performance under various conditions [7].	Using computational techniques to predict component behavior and properties, enabling virtual testing and optimization [7,28].	Zhang et al. [28], Wang et al. [30].
Machine learning	Utilizing artificial intelligence algorithms to optimize design parameters, predict material properties, and enhance overall performance in MMAM [41].	Employs various ML algorithms (e.g., neural networks, genetic algorithms) to analyze large datasets of design parameters, material properties, and performance metrics. Can be used for inverse design, property prediction, and process optimization [26].	Wang et al. [30], Teawdeswan and Dong [14], Zhang et al. [28].

**Table 6:** Material distribution strategies.

Distribution Strategy	Characteristics	Key Design Considerations	Aligned Design Approaches	Schematic Figures of Material distribution Schemes	References
Discrete Material Distribution	Clear material boundaries	Core–shell structures; Layer-wise alternation; Voxel-based methods	Topology optimization, Geometric design optimization	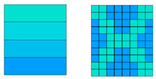	[21,26,34,44,45]
Gradient Material Distribution	Spatially varying composition	Functional gradients; Stimuli-responsive; Biological gradients	Functionally graded material design	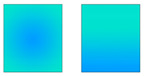	[44]
Hybrid Distribution Approaches	Combination of discrete and gradient	Controlled blending; Variable density printing	Biomimetic design, Complex material distributions	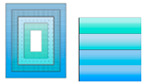	[40]
Temporal Distribution (4D Printing)	Time-dependent functionality	Smart materials; Shape-changing materials	Geometric design optimization, Biomimetic design	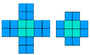	[46]

**Table 7:** Multi-material additive manufacturing processes reviewed on this paper.

Process	Key Capabilities	Limitations/Challenges	References
FFF	- Discrete and gradient distributions - Wide range of polymer combinations - Suitable for complex material combinations	- Anisotropic properties - Challenges in interface strength	[31,34,39]
LPBF	- Gradient distributions in metals - High density and strength - Process maps for property optimization	- Residual stress accumulation - Challenges in multi-material interfaces - Complex thermal management	[26,33]
DIW	- High precision for microfluidics - Suitable for biomimetic design - Stretchable multi-material structures	- Limited to specific material viscosities - Requires optimization of printing parameters - Challenges in multi-material interfaces	[23,40]
AJP	- High-precision material deposition - Suitable for electronics and sensors - AI-driven optimization potential	- Complex process parameter optimization - Requires advanced control systems - Challenges in scalability	[28]

**Table 8:** MMAM validation and characterization methods.

Process	Testing Category	Testing Method	References
FFF/FDM	Mechanical	Tensile testing, compression, flexural testing, hardness testing	[23,27,42]
FFF/FDM	Microstructural	SEM, optical microscopy	[35,52]
FFF/FDM	Non-destructive	X-ray Computed Tomography (X-CT)	[53]
FFF/FDM	Application-specific	pH responsiveness testing, shape memory tests	[28,52]
FFF/FDM	Statistical	ANOVA and regression analysis, error analysis (R², MSE, MAE)	[28,35]
LPBF	Mechanical	Microhardness and nanoindentation tests, three-point bending tests	[25,31]
LPBF	Microstructural	SEM, XRD, TEM, EDS	[25,31]
LPBF	In situ	optical monitoring	[28]
LPBF	Process-specific	Melt pool modeling	[33]
DIW	Functional	Electrical resistance measurements, conductivity detection	[42]
DIW	Process-specific	Optimization of printing parameters (nozzle size, extrusion rate, printing speed)	[43]
DIW	Application-specific	ECG signal monitoring	[43]

**Table 9:** Current methods and proposed improvements for advancing MMAM in architectural applications.

Aspect	Current Methods	Proposed Improvements
Manufacturing Tools	Most approaches are limited to homogeneous materials, primarily focusing on the load-bearing capacity of printed objects.	Adaptive printing speed and layer thicknesses to balance efficiency and quality.
Process Control	Basic material flow control with limited changes in ratios. Manual parameter adjustments based on visual inspection. Limited real-time monitoring capabilities.	Scale-up of Zhang et al.’s adaptive process control methods for building construction-scale applications [28]. Implementation of real-time feedback systems during construction [25].
Design Integration	Basic topology optimization. Separate structural and environmental performance considerations. Limited material property customization.	Simulation software for optimizing material distribution across multiple scales. Digital twin technologies based on Wang et al.’s AI-driven methods.
Digital Tools	Basic simulation models with limited multi-material capabilities. Separate design and manufacturing digital workflows.	Complex process parameter optimization. Requires advanced control systems. Challenges in scalability.
Interdisciplinary Integration	Limited integration between material selection and process parameters. Separate material and performance optimization.	Comprehensive consideration of material, process, and design parameters. Complete integration of design and manufacturing approaches.

**Table 10:** Findings and implications in multi-material additive manufacturing research.

Key Findings	Implications for Future Research in the Construction Industry	Related Studies
Topology optimization for efficient material distribution	Integrate with material selection algorithms	[20,21,42]
Multi-scale design considerations necessary	Develop multi-scale optimization approaches	[20,21,56]
Process parameters significantly influence part quality	Develop standardized optimization procedures	[23,31,32,39]
AI optimizes MMAM processes and predicts properties	Develop real-time AI control systems	[15,28,38]
Functionally graded materials achievable through design	Advance design methodologies for property gradients	[20,21,42,57]
Interfacial properties crucial for design and performance	Enhance interface design and characterization	[13,19,31,39]
Simulation crucial for process and design optimization	Advance multiphysics modeling techniques	[20,32,33]
Feeding mechanisms expand design possibilities	Develop advanced material delivery systems	[23,34]

## Data Availability

All the reviewed articles are cited in the text.
